# Differentiation of Geographical Origin of White and Brown Rice Samples Using NMR Spectroscopy Coupled with Machine Learning Techniques

**DOI:** 10.3390/metabo12111012

**Published:** 2022-10-24

**Authors:** Maham Saeed, Jung-Seop Kim, Seok-Young Kim, Ji Eun Ryu, JuHee Ko, Syed Farhan Alam Zaidi, Jeong-Ah Seo, Young-Suk Kim, Do Yup Lee, Hyung-Kyoon Choi

**Affiliations:** 1 College of Pharmacy, Chung-Ang University, Seoul 06974, Korea; 2 Department of Computer Science and Engineering, Chung-Ang University, Seoul 06974, Korea; 3 School of Systems Biomedical Science, Soongsil University, Seoul 06978, Korea; 4 Department of Food Science and Biotechnology, Ewha Womans University, Seoul 03760, Korea; 5 Center for Food and Bioconvergence, Department of Agricultural Biotechnology, Research Institute for Agricultural and Life Sciences, Seoul National University, Seoul 08826, Korea

**Keywords:** rice, geographical origin, NMR spectroscopy, machine learning, prediction model

## Abstract

Rice (*Oryza sativa* L.) is a widely consumed food source, and its geographical origin has long been a subject of discussion. In our study, we collected 44 and 20 rice samples from different regions of the Republic of Korea and China, respectively, of which 35 and 29 samples were of white and brown rice, respectively. These samples were analyzed using nuclear magnetic resonance (NMR) spectroscopy, followed by analyses with various data normalization and scaling methods. Then, leave-one-out cross-validation (LOOCV) and external validation were employed to evaluate various machine learning algorithms. Total area normalization, with unit variance and Pareto scaling for white and brown rice samples, respectively, was determined as the best pre-processing method in orthogonal partial least squares–discriminant analysis. Among the various tested algorithms, support vector machine (SVM) was the best algorithm for predicting the geographical origin of white and brown rice, with an accuracy of 0.99 and 0.96, respectively. In external validation, the SVM-based prediction model for white and brown rice showed good performance, with an accuracy of 1.0. The results of this study suggest the potential application of machine learning techniques based on NMR data for the differentiation and prediction of diverse geographical origins of white and brown rice.

## 1. Introduction

Rice (*Oryza sativa*) is a primary food source for almost 50% of the global population because of its high caloric content and various nutrients, such as minerals and vitamins [1]. Rice is an important crop in Asia and is widely consumed in various forms such as rice flour, cooked rice, and rice cookies [2]. There are two significant subspecies of *O. sativa*, indica and japonica, with an enormous number of varieties. Japonica is the most commonly cultivated crop in East Asia, particularly in Korea, Japan, and China. Genotype and environmental factors, such as rainfall intensity, soil, and temperature, heavily influence rice metabolite profiles [3]. Rice fraud has been a serious global problem. However, the assessment of botanical and geographical origin as well as cultivation methods of rice are very important [4].

Metabolomics has been employed in the field of crops and agriculture research to discriminate genetic and environmental differences, control crop quality, and determine geographical origin [5,6,7]. Various analytical platforms, such as gas chromatography–mass spectrometry, liquid chromatography/mass spectrometry, nuclear magnetic resonance (NMR) spectroscopy, Fourier-transform infrared spectroscopy, and direct-infusion mass spectrometry, have been employed to discriminate the geographical origin of crops and plants [8].

Several studies have been conducted to determine the types or geographical origins of rice samples to prevent adulteration and substitution, and to standardize their safety and quality assurance. For example, various rice samples were distinguished by multi-element fingerprinting using high-resolution inductively coupled plasma mass spectrometry (ICP-MS) [1], elemental imaging using laser ablation ICP-MS [9], and ^1^H-NMR spectroscopy coupled with principal component analysis (PCA) and discriminant analysis [10], and ^1^H-NMR spectroscopy coupled with partial least square–discriminant analysis (PLS-DA) and independent component analysis [11]. Mass spectrometry coupled with the random forest (RF) classification algorithm has also been reported to differentiate white rice samples from China and Korea [12].

Recently, machine learning (ML) algorithms have been demonstrated to significantly enhance the predictability and validity of prediction models for the geographical origin of various crops and medicinal plants. ^1^H-NMR and inductively coupled plasma atomic emission spectroscopy/ICP-MS techniques coupled with ML algorithms have been applied to discriminate the geographical origins of medicinal plants from Korea and China (*Astragalus membranaceus* and *Paeonia albiflora*) [13]. The geographical origin of 237 samples of asparagus (*Asparagus officinalis* L.) from six different countries (The Netherlands, Germany, Spain, Poland, Greece, and Peru) was successfully distinguished using ^1^H-NMR spectroscopy coupled with ML algorithms [14]. *Ixeris denticulata* samples from eight different origins were differentiated using ultra-high performance liquid chromatography-quadrupole time-of-flight mass spectrometry followed by ML algorithms [15]. Differences in soybean seed vigor were evaluated using infrared spectroscopy and machine-learning techniques [16].

The total amount of rice imported into Korea in 2021 was 492,901 tons (accounting for 12.7% of domestic production), of which 40.8% was imported from China (196,322.2 tons of brown rice, 5001.8 tons of polished rice). Most of the imported rice from China into Korea was brown rice because of its advantages in storage and variety of utilization [17,18]. It is important to distinguish the geographical origin of white and brown rice. The problem of counterfeiting the origin of imported rice is leading to economic problems as well as confusion in the domestic rice market. Over the past four years, the number of cases of illegal distribution of imported rice in the Republic of Korea has been increasing every year with about 425 cases, which gives local farmers a sense of relative deprivation and disrupts the order of the healthy rice distribution market [19]. In addition, this has affected the increase in the inventory of rice in conjunction with the overproduction of domestic rice, resulting in a surge in the amount of sales loss for feed and inventory management costs [20]. However, there have been no reports on the differentiation of the geographical origins of rice samples (with two milling types) using ^1^H-NMR analysis coupled with ML techniques.

In this study, we collected white and brown rice samples from different regions of the Republic of Korea (hereafter referred to as Korea) and China. We analyzed the data using NMR spectroscopy coupled with various ML algorithms such as PCA, orthogonal PLS-DA (OPLS-DA), random forest, decision tree, support vector machine (SVM), logistic regression, and k-nearest neighbors. The main objective of our study was to explore the utilization of NMR spectroscopy coupled with various ML algorithms to develop a convenient method for predicting the geographical origin of rice.

## 2. Materials and Methods

### 2.1. Rice Sample Collection

Rice samples, collected from Korea (44 samples) and China (20 samples) with two types of milling (white and brown rice) as shown in Supplementary Figure S1, were prepared for NMR spectroscopy analysis. Brown rice is obtained by dehusking of paddy rice, and white rice is obtained by removing the bran layer and the germ from the brown rice. Korean rice samples were harvested in 2018 and collected by the National Institute of Crop Science. The Korean rice samples were cultivated in Kangwon-do (Cheorwon, Chuncheon, Hoengseong, and Yangyang), Gyeonggi-do (Suwon, Hwaseong, Paju, and Yeoncheon), Chungcheongbuk-do (Chungju and Cheongju), Chungcheongnam-do (Asan and Seosan), Gyeongsangbuk-do (Sangju, Yecheon, and Gyeongju), Gyeongsangnam-do (Miryang and Haman), Jeollabuk-do (Jeonju, Kimje, and Jinan), and Jeollanam-do (Kangjin and Haenam). Chinese rice samples were harvested in 2018 and bought from online suppliers. These samples were obtained from Heilongjiang, Henan, Liaoning, Jilin, Jiangsu, Shandong, Shanxi, Hubei, and Sichuan provinces. The provinces, cities, and weather information for the rice samples are summarized in Supplementary Table S1 and Figure S2.

### 2.2. Chemicals and Reagents

Deuterium oxide (D_2_O, 99.9% atom D) including 0.05% 3-(trimethylsilyl) propionic-2,2,3,3-d_4_ acid sodium salt (TSP), deuterium oxide (D_2_O, 99.9% atom D), and monopotassium phosphate (KH_2_PO_4_) were purchased from Sigma-Aldrich (St. Louis, MO, USA). Sodium deuteroxide solution (NaOD, 99.5% atom D; 40% in D_2_O) was purchased from Cambridge Isotope Laboratories, Inc. (Andover, MA, USA).

### 2.3. Pre-Preparation and Extraction of Rice

Pooled samples from each location were promptly frozen in liquid nitrogen, pulverized using a blender, and stored in a deep freezer until NMR analysis. Then, 100 mg of rice powder and 1.5 mL of 100% D_2_O (0.1 mM TSP) were transferred into a 2 mL centrifuge tube (Eppendorf tube, Hamburg, Germany), vortexed for 1 min, and sonicated for 15 min. Subsequently, the suspension was centrifuged at 17,000× *g*, 4 °C for 10 min. A buffer solution of 90 mM KH_2_PO_4_ was prepared from D_2_O, and NaOD was used to adjust the pH to 6.0. The clear supernatant was filtered using a 0.45 µm PVDF filter (Chemco Scientific, Osaka, Japan), and 600 µL of the sample was transferred into a 5 mm NMR tube (Norell, Landisville, NJ, USA).

### 2.4. Peak NMR Spectra Assignment

A 600-MHz Bruker Avance spectrometer (Bruker, Germany) was employed to analyze rice samples at 25 °C to record all NMR spectra. For the ^1^H-NMR spectra, 64K data points were obtained with a relaxation delay of 2.0 s and a spectral width of 10,775.9 Hz. A total of 128 scans and an acquisition time of 3.0 s were used. Water suppression was conducted to exclude the region between δ = 4.7 and 5.0 using a pre-saturation pulse sequence (Bruker 1D noesygppr1d). For two-dimensional NMR spectra, ^1^H–^1^H correlation spectroscopy (COSY) spectra were acquired under the following conditions: 32 scans, relaxation delay of 2.0 s, and 7812.5 Hz (for white rice) and 6465.5 Hz (for brown rice) spectral widths. ^1^H–^13^C heteronuclear single quantum correlation (HSQC) spectra were obtained with 32 scans, 2.0 s relaxation delay, and spectral widths of 5122.9 Hz and 36,235.5 Hz in the F1 and F2 dimensions, respectively (for white rice), and 6465.5 Hz and 36,150.3 Hz in the F1 and F2 dimensions, respectively (for brown rice). Baseline correction and assignments of all ^1^H–NMR spectra were performed using Chenomx NMR suite software (version 8.2, Chenomx, Edmonton, AB, Canada). Metabolites were further identified based on the HMDB database (http://www.hmdb.ca/) (accessed on 2 March 2022). Non-overlapping peaks were used for peak assignment. MestReNova (version 6.0.4, Mestrelab Research, Santiago de Compostela, Spain) was employed to measure the peak J values and identify the peaks of the ^1^H–^1^H COSY and ^1^H–^13^C HSQC spectra.

### 2.5. NMR Data Pre-Processing and Measurement

Binning and normalization of the ^1^H–NMR spectral data were performed using the Chenomx NMR suite software. Baseline-corrected NMR spectral data ranging from 0.08 to 10.00 ppm were segmented into a series of small bins (total 245) with widths of 0.04 ppm, while excluding the water suppression region (4.70–4.86 ppm). The raw NMR spectral data were normalized using total area and standardized area normalization techniques. The total area normalization method was used to compute the relative intensities of the binned spectral data by dividing the spectral data by the total area of all bins. In contrast, in standardized area normalization, the relative intensities of the binned spectral data were calculated by dividing the spectral data by the area of the reference peak. Subsequently, the results of the binned datasets were converted to Microsoft Office Excel in a suitable format to quantify each compound by its loading value, and the binning values of compounds with numerous non-overlapping peaks were summed. All the pre-processed NMR spectral data with peak values were converted into comma-separated value (CSV) files for ML analysis.

### 2.6. Statistical Analysis

After normalization of the NMR data, SIMCA-P+ software (version 13.0, Umetrics, Umeå, Sweden) was used to perform multivariate statistical analysis. PCA and OPLS-DA were performed using the SIMCA software. PCA is a clustering approach that minimizes the dimensions of multivariate data, while retaining the majority of its variance without any prerequisite information about the dataset, whereas the OPLS-DA model is a supervised classification method [21]. The autofit function in the SIMCA program was used to choose the number of components such that a significant number of principal components were selected from the models.

Mean-centering was performed, unit variance (UV) and Pareto (Par) scaling were applied in both PCA and OPLS-DA, and the outcomes were compared to determine the best scaling method. The goodness-of-fit and predictability of the model were evaluated using R^2^Y and Q^2^Y parameters. The R^2^Y and Q^2^Y values are expected to be close to 1. The 10-fold cross-validation and permutation test were performed to prevent the overfitting of the model. Intercept values of R^2^Y and Q^2^Y below 0.4 and 0.05, respectively, were regarded as valid models.

### 2.7. Development of Differentiation Models and ML Algorithms

Python is a scripting language widely used in data science [22]. Differentiation models implicit in various ML algorithms were employed using the SciKit-Learn 0.24 software package. The SciKit-Learn library is a Python module that makes ML accessible to everyone and covers various supervised and unsupervised ML algorithms [23,24]. In metabolomics research, different linear and nonlinear supervised ML methods can be employed, such as OPLS-DA, logistic regression, SVM, k-nearest neighbors, decision tree, and RF. However, OPLS-DA is accepted as the gold standard among supervised algorithms because it supplies information related to contributing metabolites (variables) for group separation.

“GridSearchCV” is an algorithm in the SciKit-Learn library that selects optimal hyperparameters for each ML algorithm to identify the best differentiation model. A range of hyperparameter values can be assigned to the algorithm as inputs. The algorithm then builds models using each possible hyperparameter set from the ranges of the hyperparameters and shows the best hyperparameter settings for the selected ML algorithm. It also uses a CV method to find optimal hyperparameter values over k-fold CV [25].

Leave-one-out cross-validation (LOOCV) was used to evaluate the performance of machine learning algorithms. Most often used cross-validation techniques are k-fold and LOOCV. For larger datasets, k-fold is preferable to LOOCV. Data are split into K sets for k-fold cross-validation, with one set serving as the validation set for each iteration. In comparison, LOOCV is a special case of k-fold that employs test and training data from each sample in the dataset. LOOCV chooses one sample from the data as a validation set so that each sample can reflect the test data. However, utilizing several trained and testing models by LOOCV estimates more reliable outcomes, thus it is suitable for small datasets [26,27].

In ML algorithms, true positives (TP) are the positive classes that the model correctly classifies, and true negatives (TN) are the negative classes that are classified correctly by the model. False positives (FP) are the classes that the model incorrectly classifies as positive, and false negatives (FN) are the classes that are incorrectly classified as negative by the model [24].

To evaluate the ML algorithms, six evaluators compared the performance of the established models, including accuracy, receiver operating characteristic (ROC)–area under the curve (AUC), specificity, precision, recall, and F1_score. Accuracy measures the ratio of correctly predicted samples to the total number of samples evaluated ((TP + TN)/(TP + FP + TN + FN)) [28]. Specificity measures the fraction of negative patterns that are correctly classified (TN/(TN + FP)) [24]. Precision measures the positive patterns that are correctly predicted from the total predicted patterns in a positive class (TP/(TP + FP)) [28]. Recall measures the fraction of positive patterns that are correctly classified (TP/(TP + FN)) [24]. The F1_score is the harmonic mean of precision and recall ((2 × precision × recall)/(precision + recall)) [29]. The AUC is widely used to determine the predictability of an established model; a high AUC value represents the best performance of the model [24].

## 3. Results and Discussion

### 3.1. Identification of Metabolites in Rice

**Figure 1:**
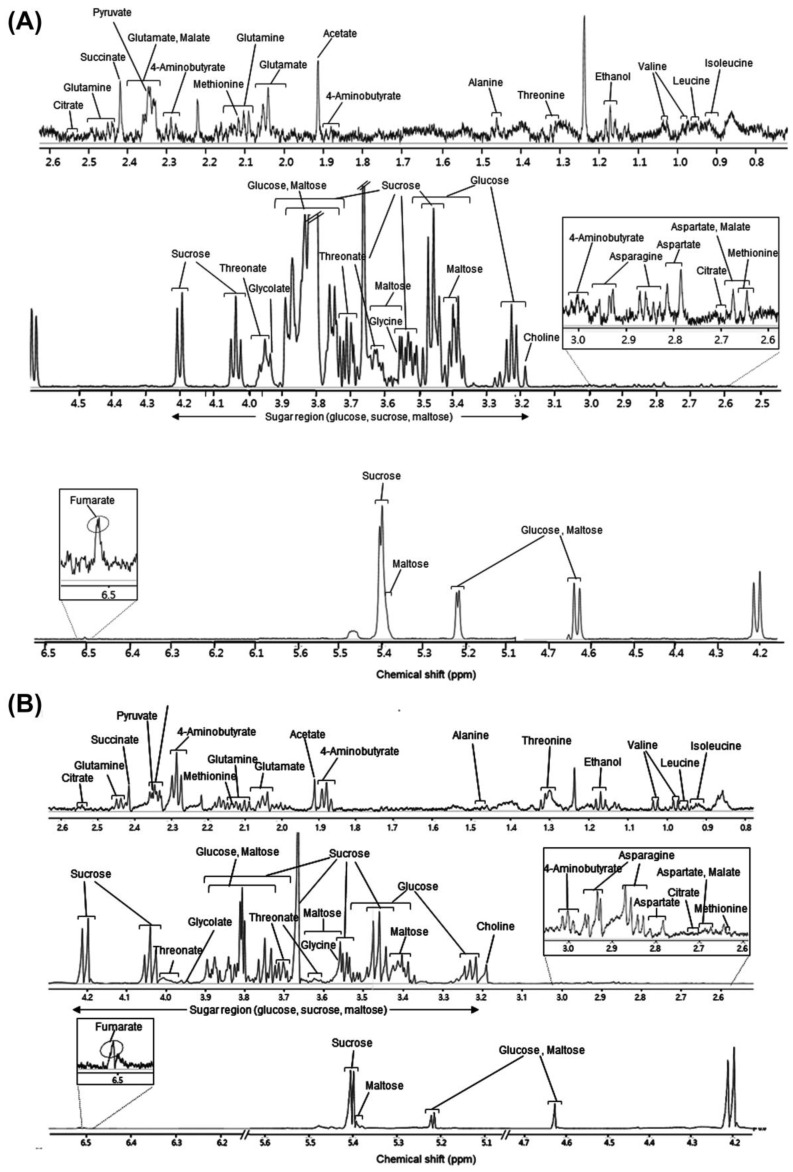
Representative one-dimensional ^1^H-nuclear magnetic resonance (NMR) spectra of white rice (**A**) and brown rice (**B**).

The putatively assigned peaks for white and brown rice are presented in Table 1, and the representative NMR spectrum for the metabolite extract is shown in Figure 1. We obtained 105 and 87 ^1^H-NMR spectra using three experimental replicates from white rice (Korea, 30 and China, 5) and brown rice (Korea, 14 and China, 15) samples, respectively.

Twenty-four metabolites, including 12 amino acids, 5 organic acids, 3 sugars, 1 alcohol, and 3 others, were identified in the rice samples using a one-dimensional NMR technique. Among the 12 amino acids, isoleucine, leucine, methionine, threonine, and valine were identified as essential amino acids. Acetate, malate, fumarate, glycolate, and succinate were found to be the organic acids. Sugars found in the rice samples included glucose, maltose, and sucrose. Two-dimensional NMR spectroscopy (COSY, HSQC) was performed to support the identification of various metabolites by one-dimensional NMR (Supplementary Figures S3 and S4).

Like other agricultural crops, metabolic profiles of rice are influenced by genotype and various environmental factors, such as rainfall, temperature, and soil [3,30]. This study considered the average rainfall and temperature in the regions from which the white and brown rice samples from Korea and China were collected. Supplementary Table S1 shows the average rainfall and temperatures of all regions. Supplementary Figure S2A,B show the average rainfall and temperature for white and brown rice collection regions in Korea and China, respectively. No significant differences in temperature were observed between Korea and China. However, a significant difference in the rainfall was observed between Korea and China. Rainfall has significant consequences for specific geographical locations and seasons. Most notably, during critical phases of crop growth, protracted durations of rainfall may drain considerable amounts of essential substances from plants, such as amino acids, organic acids, and polysaccharides [31]. Rainfall and solar radiation have been reported to significantly affect tea production and quality [32]. In addition, in most plants, a change in one factor can affect the metabolite content, even when other factors remain constant [33]. Therefore, we speculate that rainfall was the main environmental factor responsible for the differences in the metabolites of rice samples.

### 3.2. PCA Model Establishment for Predicting the Geographical Origin of Rice

**Figure 2:**
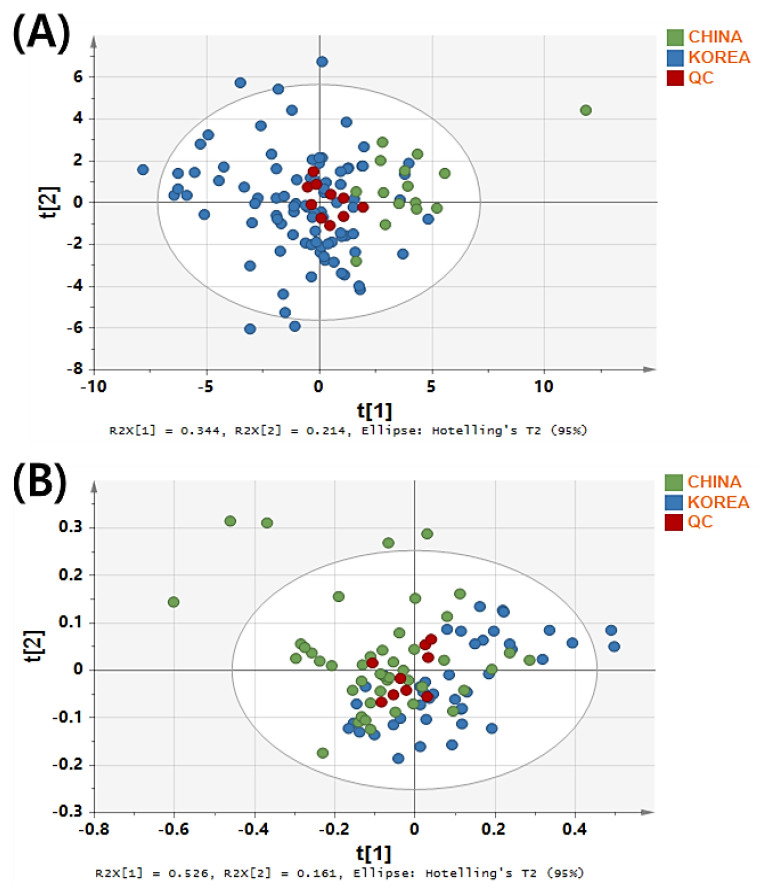
Principal component analysis (PCA) score plots for discriminating the geographical origin of white rice (**A**) and brown rice (**B**) samples from Korea and China.

In this study, PCA was used to objectively analyze the ^1^H-NMR data. In the PCA score plot (Figure 2), the white and brown rice samples from Korea and China were distinctly separated by partial conjoining. The UV and Par scaling methods for white and brown rice, respectively, showed better clustering of 10 quality control (QC) samples, demonstrating the instrumental stability and reliability of NMR spectroscopy. For white rice, the principal components (PC1 and PC2) collectively accounted for 55.8% of the total variation, with R^2^X = 0.975 and Q^2^ = 0.607. For brown rice, the principal components (PC1 and PC2) collectively accounted for 68.7% of the total variation, with values of R^2^X = 0.984, and Q^2^ = 0.692.

### 3.3. Comparing ML Models for Predicting the Geographical Origin of Rice

**Figure 3:**
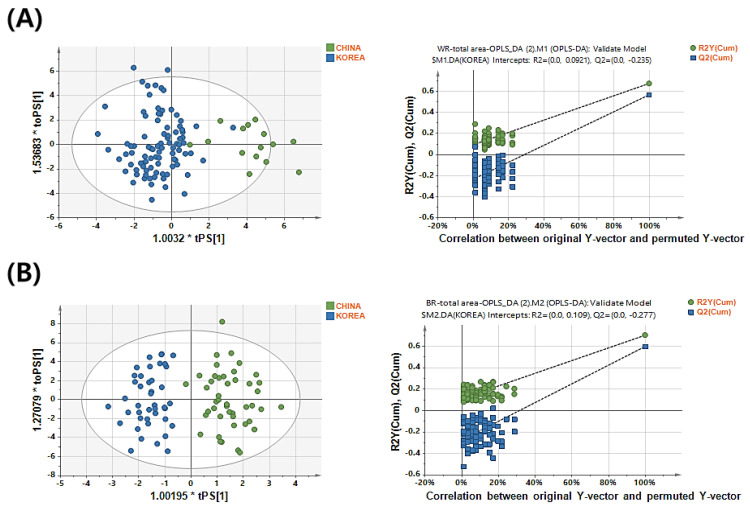
Orthogonal partial least square–discriminant analysis (OPLS-DA) score plots and permutation test plots for discriminating the geographical origin of white rice (**A**) and brown rice (**B**) samples from Korea and China.

Table 2 lists the model performance (R^2^Y and Q^2^Y) and parameters (intercept values of R^2^Y and Q^2^Y) of permutation for predicting Korean and Chinese white and brown rice samples with different normalization and scaling methods. For white rice samples, the model established with total area normalization and UV scaling showed the highest R^2^Y and Q^2^Y values, 0.673 and 0.566, respectively. Therefore, we selected this model as the optimal model. The permutation test was also satisfied with R^2^Y and Q^2^Y intercept values of 0.0731 and −0.196, respectively. OPLS-DA-derived score plots showed an explicit separation between the Korean and Chinese rice samples (Figure 3A).

For brown rice samples, the total area normalization and Par scaling methods were optimal with satisfactory R^2^Y and Q^2^Y values of 0.702 and 0.597, respectively (Table 2). The score plots showed an explicit separation between the Korean and Chinese rice samples (Figure 3B). The permutation test also yielded R^2^Y and Q^2^Y intercept values of 0.119 and −0.275, respectively (Figure 3B, Table 2). The OPLS-DA-derived score plots also showed a clear separation between the Korean and Chinese rice samples (Figure 3B). The model with total area normalization and UV scaling showed the highest value closest to 1 for brown rice; however, it showed scattered plots of QC samples in the PCA-derived score plots. The clustering of QC samples represents the robustness and reproducibility of the analysis [34,35]. Therefore, total area normalization and Par scaling were selected for discrimination of the Korean and Chinese brown rice samples because they showed better clustering of the QC samples than that with the other methods.

To distinguish between Chinese and Korean rice, the total area normalization method, which divided each metabolite peak area by the total peak area, was used, giving each sample the same total peak area of 1. The total area normalization method is one of the most widely used normalization techniques for NMR data in metabolomics research. Consequently, each peak intensity can be reported as a percentage of the total peak intensity, making it possible to compare metabolite levels across samples in the same unit [36,37]. By assigning equal values to each metabolite and setting the standard deviation to one for all metabolites, the UV scaling strategy is one of the simplest ways to normalize metabolic variability [37,38,39]. Appropriate normalization and scaling techniques are crucial for improving the biological information in metabolomics data. These techniques decrease unwanted biases induced by biological and technical variance and compensate for different ranges between samples or variables for comparison [37,40,41]. Normalization considerably decreases metabolite intensity variance between samples (sample-to-sample variance), allowing all samples to be compared. Scaling balances intensity variation between metabolites (metabolite-to-metabolite comparison), allowing all metabolites to be compared [36,37].

Table 3 presents the comparison of the performance of the differentiation models established by various ML algorithms after LOOCV. The SVM-based differentiation model outperformed the RF-, decision tree-, k-nearest-neighbors-, and OPLS-DA-based differentiation models. The mentioned parameters were selected using “GridSearchCV”. SVM is prone to overfitting; however, the correct type of kernel selection makes it robust to noise and overfitting [14,42]. The SVM can handle outliers efficiently because it uses a maximum margin solution. The maximum margin solution uses a maximum margin separating hyperplane for optimization [43]. SVM has a significant advantage over PLS and OPLS because the SVM model can be built using both linear and nonlinear kernels [42].

We employed datasets obtained from the ^1^H-NMR analysis, which are not high-dimensional because the number of features is less than the number of samples. High-dimensional data correspond to data with more features than the number of samples [44]. As discussed previously, the choice of kernel is essential. Therefore, “GridSearchCV” was used with different kernels and parameter sets to find the best kernel type to distinguish the rice samples. In our case, the SVM model with a linear kernel showed better train and test accuracy than the radial basis function and polynomial and sigmoid kernels. Kernel type may differ for different ^1^H-NMR data for other crops.

The linear SVM classified rice samples using a single line or hyperplane. A line or hyperplane is adjusted by updating weights or intercept values during training. The SVM finds the best weights and intercept values that create a hyperplane or decision boundary [45,46]. The closest samples to the decision boundary were identified as support vectors, which draw lines parallel to the decision boundary to provide an optimized solution, called the maximum margin solution [47,48].

Furthermore, SVM can perform better than other standard classification algorithms. Logistic regression is also a linear classifier that can classify data by a hyperplane; however, the activation function makes it different from SVM because logistic regression uses a sigmoid function instead of a maximum margin solution [49], which does not give an optimal solution. However, the logistic regression algorithm requires a large sample size for better and stable model training and shows poor performance with irrelevant and highly correlated data. The decision tree algorithm uses Gini or information gain for building the tree. The bias of the decision tree is to find the smallest tree that can classify the data. If data change slightly or are noisy, the outcomes can vary considerably.

Moreover, decision tree algorithm cannot deal with high-dimensional data and can easily be overfitted to training data [50]. Random forest algorithms ensemble many trees during training [51], slowing down the algorithms by increasing the number of trees. Moreover, the predictions of the trees need to be uncorrelated [50]. KNN is time-consuming for large datasets and requires data scaling because it uses distance for finding neighbors, is sensitive to outliers [49], cannot handle missing values, and does not work well for imbalanced datasets [49,50].

In comparison, SVM can perform better than other standard classification algorithms for imbalanced datasets [52]. The imbalance dataset has significantly more samples than other classes, which is a cause of model overfitting. However, for our rice dataset, SVM’s optimal nature showed better performance than other standard classifiers because it can handle both balanced (brown rice dataset; Korea, 14 and China, 15) and imbalanced datasets (white rice dataset; Korea, 30 and China, 5).

UV-scaled data were used for white rice to establish the model, and the best differentiation model was developed by applying SVM (accuracy and ROC-AUC of 0.99 in the test set). Par-scaled data were used for brown rice to establish the model, and the best differentiation model was developed by applying an SVM (accuracy and ROC-AUC of 0.96 in the test set). AUC values of 0.99 (for white rice) and 0.96 (for brown rice) were determined through the ROC curve analysis for predicting the geographical origin of rice, which suggested that the discovered 24 metabolites might be utilized to distinguish between rice samples from Korea and China. Thus, the SVM model developed in this study can be used to distinguish and predict Korean and Chinese rice samples.

In total, 105 and 87 rice sample spectra were analyzed using NMR. SVM showed better performance than other ML algorithms, as it offers great generalization ability for a small sample size [53,54]. Generalization is a ML term, which means that the model should be able to make appropriate decisions for unseen data based on previously observed data [55]. Hou et al. [53] identified 11 types of edible oil (from 52 samples) by employing an SVM based on a low-field nuclear magnetic resonance dataset comprising five extracted features.

Table 4 presents the performance of the SVM model in discriminating between white and brown rice using external validation. When establishing a differentiation model, internal validation is essential; however, external validation is also suggested to acquire important information regarding the existing or previously developed performance of the model [56]. External validation was performed using the previously selected SVM method by importing validation samples. For white rice, the entire dataset (*n* = 105) was randomly divided into development (*n* = 90) and validation (*n* = 15) samples. For brown rice, the entire dataset (*n* = 87) was randomly divided into development (*n* = 74) and validation (*n* = 13) samples. The performance scores (white and brown rice) were 0.96 or higher for the development set, whereas they were 1.0 for the validation set.

Compared with mass spectrometry (MS)-based metabolic profiling, NMR-based metabolic profiling has advantages in rapid sample preparation and higher reproducibility [57]. However, it has lower sensitivity than MS-based metabolic profiling. Thus, it is suggested that both MS- and NMR-based metabolic profiling be employed as complementary methods for the discrimination of various crops including rice. In a previous report, MS-based metabolic profiling coupled with PLS-DA and random forest models discriminated the geographical origin of white rice samples [12]. In our study, we established optimal normalization and scaling methods for NMR datasets to differentiate white and brown rice samples from Korea and China. We also found that the SVM applied to NMR-based metabolic profiles outperforms the PLS-DA and random forest in predicting the geographical origins of white and brown rice from Korea and China. In particular, differentiation of geographical origins of brown rice was conducted for the first time in our study using NMR-based metabolic profiling.

The limitation of machine learning techniques compared with the widely used multivariate statistical analyses, such as PLS-DA or OPLS-DA, is the lack of information about the contributing factors (metabolites) for the differentiation of each group. The machine learning techniques should be employed when the main aim is the practical differentiation or prediction, rather than revelation of contributing factors.

For practical use of established methods in this study, the expensive cost of the NMR equipment and its maintenance should be considered, especially in developing countries. Establishment and effective management of a nationwide centralized laboratory system can be a promising approach for the high-cost problem. In future studies, an extensive sample collection and analysis could be performed to establish a robust differentiation model for discriminating rice samples from various countries worldwide.

## 4. Conclusions

This is the first study to discriminate the geographical origin of rice from Korea and China with two milling types (white and brown) using NMR spectroscopy coupled with the most widely used ML algorithms. The SVM-based classification showed the best results in the LOOCV and external validation of the white and brown rice samples. This study can be employed as a complementary and alternative approach to previously reported analytical techniques for the geographical discrimination of rice samples. The concept and results of this study could be used for establishing a robust model for differentiation of rice samples from various countries worldwide in future studies.

## Figures and Tables

**Table 1:** Putative peak assignment of nuclear magnetic resonance (NMR) spectra in rice.

No.	Compound	InChI Key	Chemical Shift	Assignment Method	
			(Multiplicity ,J Value)	White Rice	Brown Rice
	**Amino Acids**				
1	4-Aminobutyrate	BTCSSZJGUNDROE-UHFFFAOYSA-N	1.84–1.92 (m), 2.29 (t, J = 7.4), 3.00 (t, J = 7.2)	1D	1D, HSQC
2	Alanine	QNAYBMKLOCPYGJ-REOHCLBHSA-N	1.47 (d, J = 7.2)	1D	1D
3	Asparagine	DCXYFEDJOCDNAF-REOHCLBHSA-N	2.80–2.92 (m), 2.88–3.00 (m)	1D	1D
4	Aspartate	CKLJMWTZIZZHCS-REOHCLBHSA-N	2.80 (dd, J = 17.4, 3.9)	1D, COSY	1D
5	Glutamate	WHUUTDBJXJRKMK-UHFFFAOYSA-N	2.00–2.10 (m)	1D, COSY	1D, COSY
6	Glutamine	ZDXPYRJPNDTMRX-VKHMYHEASA-N	2.06–2.20 (m), 2.38–2.50 (m)	1D	1D
7	Glycine	DHMQDGOQFOQNFH-UHFFFAOYSA-N	3.56 (s)	1D	1D
8	Isoleucine	AGPKZVBTJJNPAG-WHFBIAKZSA-N	0.92 (t, J = 7.2), 1.00 (d, J = 7.2)	1D, HSQC	1D, COSY, HSQC
9	Leucine	ROHFNLRQFUQHCH-YFKPBYRVSA-N	0.95 (t, J = 6.2)	1D	1D, COSY
10	Methionine	FFEARJCKVFRZRR-BYPYZUCNSA-N	2.66 (t, J = 7.8)	1D, COSY	1D, COSY
11	Threonine	AYFVYJQAPQTCCC-GBXIJSLDSA-N	1.31 (d, J = 6.6)	1D, COSY	1D, COSY
12	Valine	KZSNJWFQEVHDMF-BYPYZUCNSA-N	0.98 (d, J = 6.9), 1.03 (d, J = 6.6)	1D	1D, COSY
	**Organic acids**				
13	Malate	BJEPYKJPYRNKOW-UHFFFAOYSA-N	4.33 (d, J = 7.8)	1D, COSY	1D, COSY
14	Fumarate	VZCYOOQTPOCHFL-OWOJBTEDSA-N	6.51 (s)	1D	1D
15	Succinate	KDYFGRWQOYBRFD-UHFFFAOYSA-N	2.42 (s)	1D	1D
16	Acetate	QTBSBXVTEAMEQO-UHFFFAOYSA-M	1.91 (s)	1D	1D
17	Glycolate	AEMRFAOFKBGASW-UHFFFAOYSA-N	3.95 (s)	1D	1D
	**Sugars**				
18	Glucose	WQZGKKKJIJFFOK-GASJEMHNSA-N	4.63 (d, J = 7.8), 5.22 (d, J = 3.6)	1D, HSQC	1D, HSQC
19	Maltose	GUBGYTABKSRVRQ-PICCSMPSSA-N	5.41 (d, J = 3.6)	1D, COSY, HSQC	1D, COSY, HSQC
20	Sucrose	CZMRCDWAGMRECN-UGDNZRGBSA-N	3.46 (t, J = 9.6), 3.67 (s), 3.75 (t, J = 9.6), 4.04 (t, J = 8.6), 4.21 (d, J = 8.7), 5.39 (d, J = 3.6)	1D, COSY, HSQC	1D, COSY, HSQC
	**Alcohol**				
21	Ethanol	LFQSCWFLJHTTHZ-UHFFFAOYSA-N	1.17 (t, J = 7.2)	1D	1D
	**Others**				
22	Pyruvate	LCTONWCANYUPML-UHFFFAOYSA-N	2.36 (s)	1D	1D
23	Threonate	JPIJQSOTBSSVTP-STHAYSLISA-N	4.00 (d, J = 2.4)	1D, HSQC	1D, HSQC
24	Choline	OEYIOHPDSNJKLS-UHFFFAOYSA-N	3.19 (s)	1D, HSQC	1D, HSQC

s, singlet; d, doublet; dd, doublet of doublets; t, triplet; q, quartet; m, multiplet; 1D, 1-dimensional; COSY, correlation spectroscopy; HSQC, heteronuclear single quantum correlation.

**Table 2:** Orthogonal partial least square–discriminant analysis (OPLS-DA) model parameters based on various normalization and scaling methods for discriminating the geographical origin of rice samples.

Group No.	Normalization Method	Scaling Method	Component Number	R^2^Y	Q^2^Y	R^2^Y Intercept	Q^2^Y Intercept
**White Rice**							
1	Total area	UV	1 + 3 + 0	0.673	0.566	0.0731	−0.196
2		Par	1 + 3 + 0	0.623	0.538	0.0941	−0.244
3	Standardized area	UV	1 + 1 + 0	0.396	0.233	0.0276	−0.292
4		Par	-	-	-	-	-
**Brown rice**							
1	Total area	UV	1 + 7 + 0	0.844	0.736	0.172	−0.403
2		Par	1 + 4 + 0	0.702	0.597	0.119	−0.275
3	Standardized area	UV	1 + 6 + 0	0.827	0.723	0.144	−0.386
4		Par	1 + 7 + 0	0.82	0.702	0.152	−0.399

UV, unit variance; Par, pareto.

**Table 3:** Comparison of leave-one-out cross-validation (LOOCV) performance of various machine learning algorithms for discriminating the geographical origin of white and brown rice from Korea and China.

**White Rice**	**Parameters**	**Accuracy**		**ROC-AUC**		**Specificity**		**Precision**		**Recall**		**F1_Score**	
**Methods**		**Train**	**Test**	**Train**	**Test**	**Train**	**Test**	**Train**	**Test**	**Train**	**Test**	**Train**	**Test**
Random forest	criterion = ‘gini’ max_depth = 4, min_samples_leaf = 2,	0.94	0.92	0.83	0.78	0.99	0.98	0.94	0.92	0.94	0.92	0.94	0.92
	min_samples_split = 20, random state = 0												
	n_estimators = 10												
Decision tree	criterion = ‘gini’ max_depth = 2, random state=0	0.99	0.91	0.95	0.81	0.99	0.96	0.99	0.91	0.99	0.91	0.99	0.91
SVM	C = 3, gamma = 0.01, kernel = ‘linear’	1.00	0.99	1.00	0.99	1.00	0.99	1.00	0.99	1.00	0.99	1.00	0.99
Logistic regression	C = 2, max_iter = 100,’	1.00	0.96	1.00	0.89	1.00	0.99	1.00	0.96	1.00	0.96	1.00	0.96
	random_state = 0, solver = ‘lbfgs												
KNN	n_neighbors = 2, weights = ‘distance’	1.00	0.97	1.00	0.96	1.00	0.98	1.00	0.97	1.00	0.97	1.00	1.97
OPLS-DA	components = 1 + 3 + 0	0.96	0.98	0.98	0.98	0.99	0.98	0.95	0.96	0.89	0.89	0.92	0.91
**Brown rice**	**Parameters**	**Accuracy**		**ROC-AUC**		**Specificity**		**Precision**		**Recall**		**F1_score**	
**Methods**		**Train**	**Test**	**Train**	**Test**	**Train**	**Test**	**Train**	**Test**	**Train**	**Test**	**Train**	**Test**
Random forest	criterion = ‘entropy’ max_depth = 3, min_samples_leaf = 2,	0.99	0.92	0.98	0.92	1.00	0.95	0.99	0.92	0.99	0.92	0.99	0.92
	min_samples_split = 10, random state = 0												
	n_estimators = 20												
Decision tree	criterion = ‘gini’ max_depth = 2, random state = 0	0.98	0.94	0.99	0.94	0.98	0.95	0.98	0.94	0.98	0.94	0.98	0.94
SVM	C = 250, kernel = ‘linear’	1.00	0.96	1.00	0.96	1.00	0.96	1.00	0.95	1.00	0.95	1.00	0.95
Logistic regression	C = 2, max_iter = 10,’	0.83	0.78	0.82	0.78	0.82	0.78	0.83	0.78	0.83	0.78	0.82	0.78
	random_state = 0, solver = ‘liblinear’												
KNN	n_neighbors = 6, weights = ‘distance’	1.00	0.91	1.00	0.92	1.00	0.93	1.00	0.91	1.00	0.91	1.00	0.91
OPLS-DA	components = 1 + 4 + 0	0.98	0.95	1.00	0.96	0.99	0.98	0.99	0.98	0.97	0.94	0.98	0.96

SVM, LR, RF, KNN, and DT were performed using Scikit-Learn software, and the parameters were selected by the “GridSearchCV” function in SciKit-Learn. OPLS-DA was performed using SIMCA software, and the parameters were selected by the “Autofit” function in SIMCA software. DT, decision tree; KNN, k-nearest neighbors; LR, logistic regression; OPLS-DA, orthogonal partial least squares–discriminant analysis; RF, random forest; SVM, support vector machine.

**Table 4:** Prediction performance of the support vector machine (SVM)-based machine learning model to discriminate the geographical origin of rice from the development and external validation datasets.

Evaluators	White Rice (SVM)			Brown Rice (SVM)		
	Developmental Model (*n* = 90)		Validation Model(*n* = 15)	Developmental Model (*n* = 74)		Validation Model (*n* = 13)
	Train	Test		Train	Test	
Accuracy	1.00	0.97	1.00	1.00	0.96	1.00
ROC-AUC	1.00	1.00	1.00	1.00	1.00	1.00
Specificity	1.00	0.96	1.00	1.00	1.00	1.00
Precision	1.00	0.97	1.00	1.00	0.96	1.00
Recall	1.00	0.97	1.00	1.00	0.96	1.00
F1_score	1.00	0.97	1.00	1.00	0.96	1.00

## Data Availability

The data that support the findings of this study are available from the corresponding author upon reasonable request.

## Supplementary Materials

The following supporting information can be downloaded at: https://www.mdpi.com/article/10.3390/metabo12111012/s1, Figure S1. Map showing the origin of Korean (A) and Chinese (B) rice samples used in the experiment; Figure S2. Climate data for white rice (A) and brown rice (B) samples from Korea and China; Figure S3. Two-dimensional NMR spectra of white rice samples. (A) 1H-1H COSY spectrum and (B) 1H-13C HSQC spectrum; Figure S4. Two-dimensional NMR spectra of brown rice samples. (A) 1H-1H COSY spectrum and (B) 1H-13C HSQC spectrum; Table S1. The provinces, cities, and weather information (year: 2018) of rice samples from the Republic of Korea and China.

## Abbreviations

AUC	area under the curve
COSY	correlation spectroscopy
HMDB	Human Metabolome Database
HSQC	heteronuclear single quantum correlation
NMR	nuclear magnetic resonance
OPLS-DA	orthogonal partial least square–discriminant analysis
Par	Pareto
PCA	principal component analysis
PLS-DA	partial least square–discriminant analysis
RF	random forest
ROC	receiver operating characteristic
SVM	support vector machine
UV	unit variance
