# Supplementary material for: Differentiation of Geographical Origin of White and Brown Rice Samples Using NMR Spectroscopy Coupled with Machine Learning Techniques
Source: Metabolites. 2022 Oct 24;12(11):1012. doi: 10.3390/metabo12111012 (PMC9693418; doi:10.3390/metabo12111012)
Supplement: Supplementary file 1 [file metabolites-12-01012-s001.zip › metabolites-1904076-supplementary.pdf]

Differentiation of Geographical Origin of White and Brown Rice Samples Using NMR Spectroscopy Coupled with Machine Learning Techniques

Spplementary Information

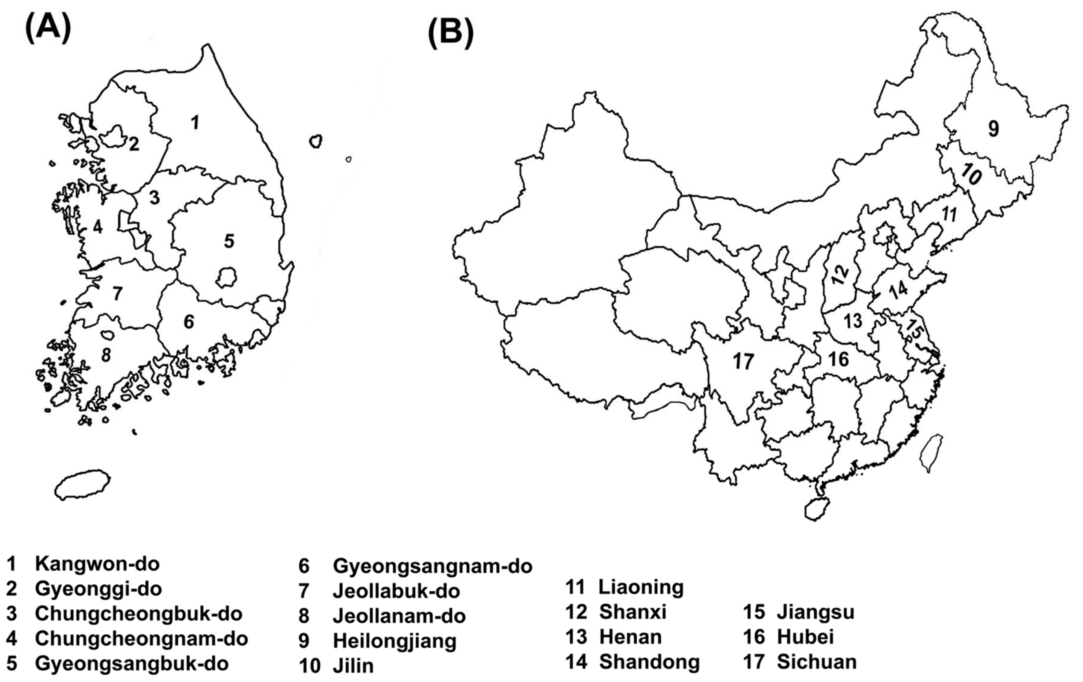

Figure S1. Map showing the origin of Korean (A) and Chinese (B) rice samples used in the experiment.

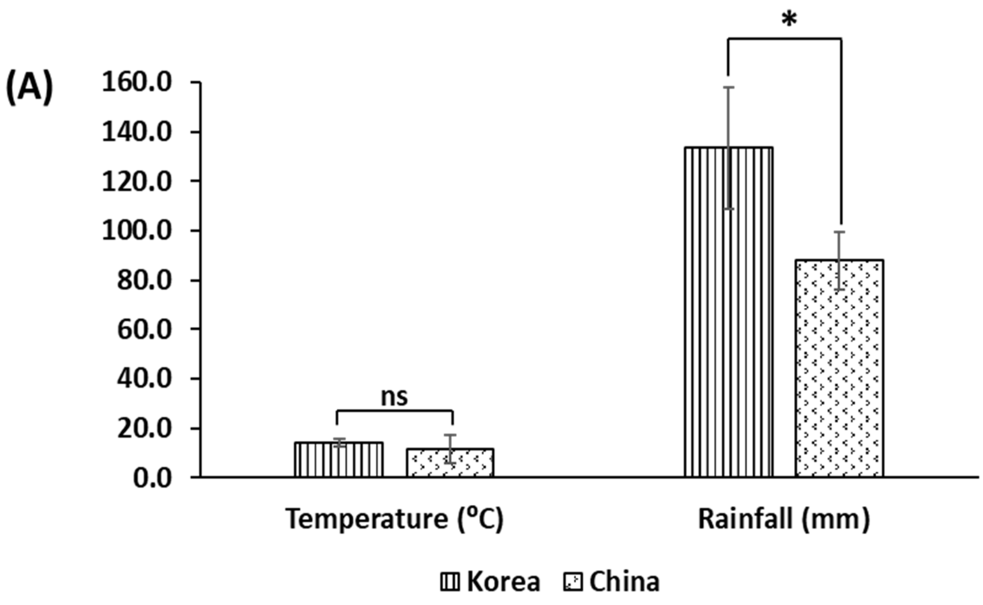

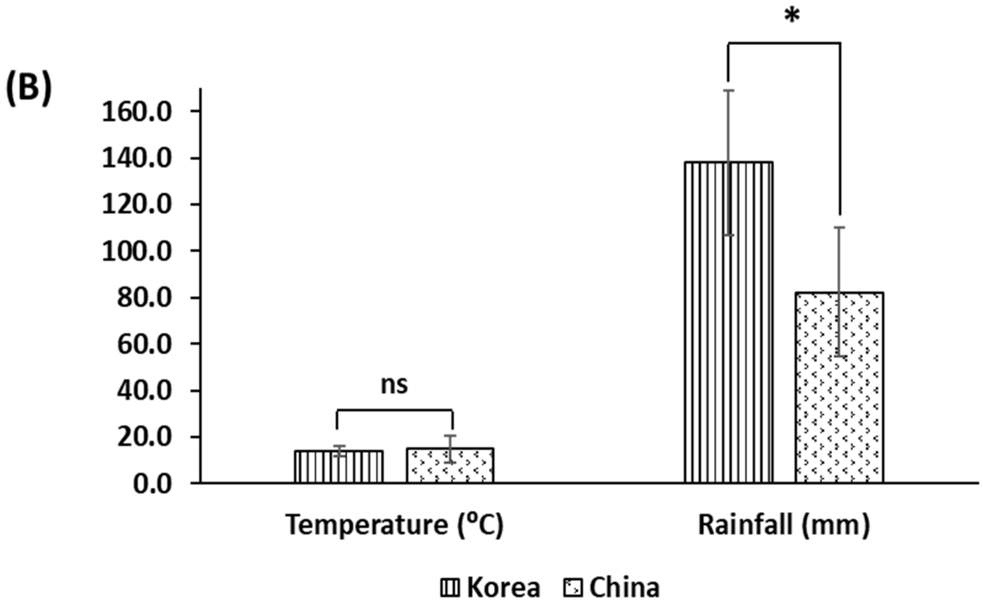

Figure S2. Climate data for white rice (A) and brown rice (B) samples from Korea and China.

Comparison of white rice (A) temperature by *t-test* ($p<0.05$) due to unequal distribution of samples, and rainfall by Mann Whitney test ($p<0.05$). Comparison of brown rice (B) temperature and rainfall by Mann Whitney test ($p<0.05$). The asterisk mark indicates significant differences between samples. NS means not significant.

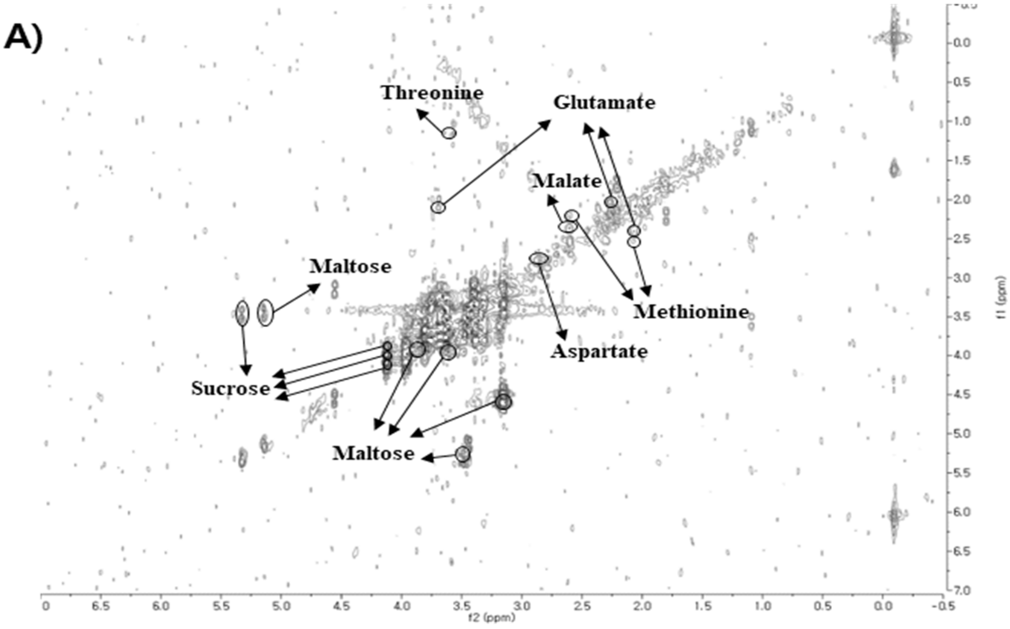

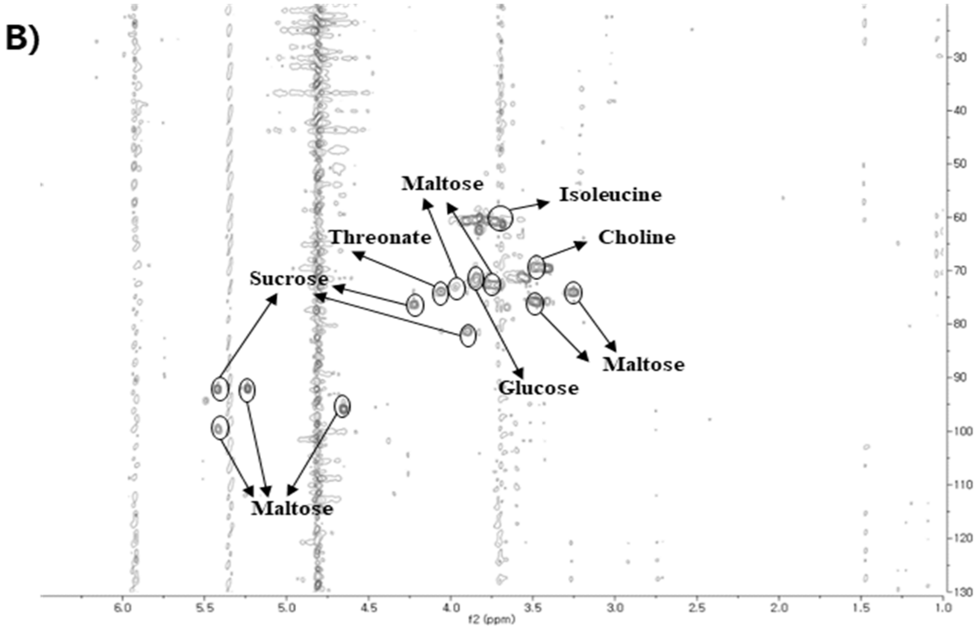

Figure S3. Two-dimensional NMR spectra of white rice samples. (A) ^{1}H-^{1}H COSY spectrum and (B) ^{1}H-^{13}C HSQC spectrum.

COSY, correlation spectroscopy; HSQC, heteronuclear single quantum correlation.

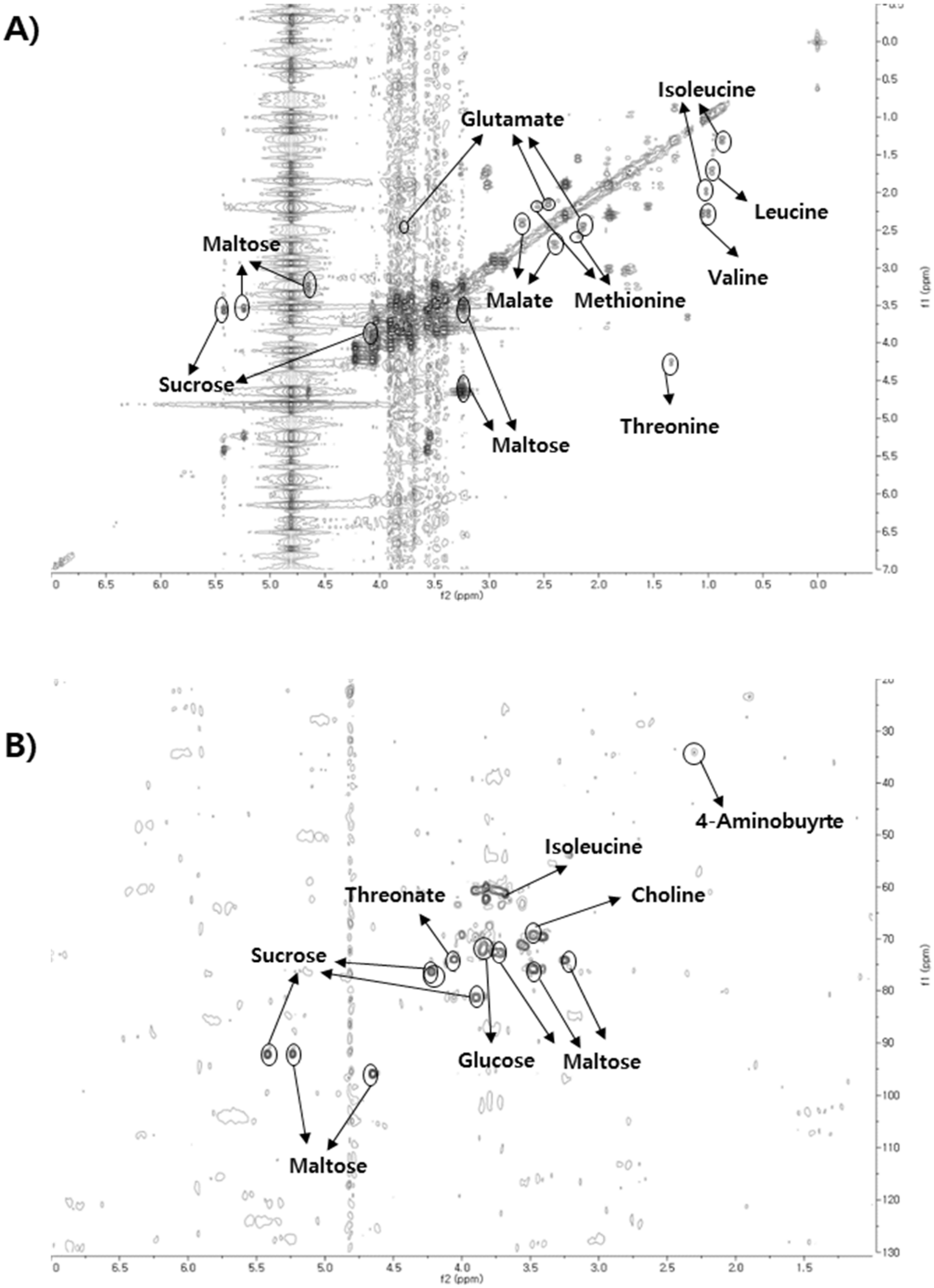

Figure S4. Two-dimensional NMR spectra of brown rice samples.

(A) ^{1}H-^{1}H COSY spectrum and (B) ^{1}H-^{13}C HSQC spectrum.

COSY, correlation spectroscopy; HSQC, heteronuclear single quantum correlation.

Table S1. The provinces, cities, and weather information (year: 2018) of rice samples from The Republic of Korea and China.

Country	Region: Province	Region: City	Avg Temp (ºC)	Avg Rainfall (mm)
White rice				
Korea	Kangwon-do	Cheorwon	10.8[c]	117.36[c]
		Chuncheon	11.75[a]	155.75[a]
		Hoengseong	9.8[b]	-
		Yangyang	12.17[a]	134.43[a]
	Gyeonggi-do	Suwon	17[a]	177.32[a]
		Hwaseong	13.44[b]	-
		Paju	13.92[a]	94.43[a]
		Yeoncheon	13.59[b]	-
	Chungcheongbuk-do	Cheongju	14.58[a]	139.19 [a]
		Chungju	13.33[a]	129.37[a]
	Chungcheongnam-do	Asan	14.17[a]	131.75[a]
		Seosan	14.33[a]	136.75[a]
	Gyeongsangbuk-do	Sangju	13.5[a]	108.12[a]
		Yecheon	13.59[b]	-
		Gyeongju	15 [a]	100.06[a]
	Gyeongsangnam-do	Miryang	14.42[a]	151.53[a]
		Haman	14.25[a]	155.24[a]
	Jeollabuk-do	Jeonju	15.17[a]	95.44[a]
		Kimje	15.17[a]	164.09[a]
		Jinan	15.16[b]	-
	Jeollanam-do	Kangjin	15.58[a]	146.53[a]
		Haenam	15.58[a]	151.54[a]
China	Heilongjiang	Hegang	5.58[a]	75.87[a]
		Harbin	7.25[a]	77.1[a]
	Jilin	Yanbian	-	-
	Liaoning	Huludao	11.83[a]	50.53[a]
	Henan	Xinxiang	18[a]	80.26[a]
Brown rice				
Korea	Kangwon-do	Cheorwon	9.86[b]	-
		Chuncheon	11.75[a]	155.75[a]

	Gyeonggi-do	Suwon	17[a]	177.32[a]
		Hwaseong	13.44[b]	-
	Chungcheongbuk-do	Cheongju	14.58[a]	139.19[a]
	Gyeongsangbuk-do	Sangju	13.5[a]	108.12[a]
	Gyeongsangnam-do	Miryang	14.42[a]	151.53[a]
	Jeollabuk-do	Jeonju	15.17[a]	95.44[a]
China	Heilongjiang	Hegang	5.58[a]	75.87[a]
		Shuangyashan	5.75[a]	84.99[a]
		Jiamusi	5.83[a]	77.59[a]
		Harbin	7.25[a]	77.1[a]
	Liaoning	Huludao	11.83[a]	50.53[a]
	Shanxi	Changzhi	13.83[a]	47.18[a]
	Henan	Nanyang	19.08[a]	62.28[a]
		Xinxiang	18[a]	80.26[a]
		Shangqiu	18.92[a]	77.87[a]
	Shandong	Zaozhuang	17.83[a]	69.13[a]
		Linyi	18.08[a]	64.48[a]
		Taian	17.33[a]	83.87[a]
	Jiangsu	Taizhou	20.42[a]	153.61[a]
	Hubei	Huanggang	21.08[a]	102.62[a]
	Sichuan	Nanchong	20.58[a]	126.33[a]

a, https://www.worldweatheronline.com/

b, South Korea Climate Zone, Weather By Month and Historical Data

c, Weather Online UK - current weather and weather forecast worldwide
